# Contribution of Oligodendrocytes, Microglia, and Astrocytes to Myelin Debris Uptake in an Explant Model of Inflammatory Demyelination in Rats

**DOI:** 10.3390/cells12172203

**Published:** 2023-09-03

**Authors:** Mariarosaria Cammarota, Francesca Boscia

**Affiliations:** Division of Pharmacology, Department of Neuroscience, Reproductive Sciences and Dentistry, School of Medicine, Federico II University of Naples, 80131 Naples, Italy; mariarosaria.cammarota@unina.it

**Keywords:** neuroinflammation, myelin, myelin uptake, OPCs, oligodendrocytes, astrocytes, microglia

## Abstract

The internalization and degradation of myelin in glia contributes to the resolution of neuroinflammation and influences disease progression. The identification of a three-dimensional experimental model to study myelin processing under neuroinflammation will offer a novel approach for studying treatment strategies favoring inflammation resolution and neuroprotection. Here, by using a model of neuroinflammation in hippocampal explants, we show that myelin debris accumulated immediately after insult and declined at 3 days, a time point at which tentative repair processes were observed. Olig2^+^ oligodendrocytes upregulated the LRP1 receptor and progressively increased MBP immunoreactivity both at peri-membrane sites and within the cytosol. Oligodendrocyte NG2^+^ precursors increased in number and immunoreactivity one day after insult, and moderately internalized MBP particles. Three days after insult MBP was intensely coexpressed by microglia and, to a much lesser extent, by astrocytes. The engulfment of both MBP^+^ debris and whole MBP^+^ cells contributed to the greatest microglia response. In addition to improving our understanding of the spatial-temporal contribution of glial scarring to myelin uptake under neuroinflammation, our findings suggest that the exposure of hippocampal explants to LPS + IFN-γ-induced neuroinflammation may represent a valuable demyelination model for studying both the extrinsic and intrinsic myelin processing by glia under neuroinflammation.

## 1. Introduction

A common pathological alteration in various inflammatory and central nervous system (CNS) diseases, such as multiple sclerosis (MS), is the damage to myelin and the generation of myelin debris resulting from the breakdown of the axonal myelin sheath [1,2]. Beyond the functional implication of the loss of axonal myelin coverage on action potential propagation, it is now clear that over-accumulation of myelin-toxic debris contributes to chronic inflammation, failure of remyelination, and prevention of neuronal recovery [3]. Myelin debris clearance is a prerequisite for healing in CNS disorders [4,5]; microglia/macrophages are key professional phagocytes both in development and in pathology, as they recognize, engulf, and digest both cell debris and degenerated myelin [6,7,8]. Other semiprofessional phagocytes, including astrocytes and oligodendrocytes, have also been shown to participate in myelin processing in different CNS physiological and pathological states [9,10,11].

A better understanding of the mechanism regulating myelin debris degradation in time and space is required for the identification of novel pharmacological approaches to encourage reparative processes and functional recovery in the injured CNS. In this regard, the characterization of an ex vivo experimental model for studying different aspects of myelin processing is required for the identification of novel myelin clearance-enhancing therapeutics. Therapeutic screens in organotypic cultures prepared from rodent postnatal brains provide a valuable means of studying both the neuroprotective actions of compounds and their mechanism of action in a multicellular CNS context [12,13]. They preserve the three-dimensional architecture of the brain, neuro-glia networks, and synaptic organization, and, compared to in vivo approaches, are more accessible to pharmacological manipulations.

The exposure of organotypic explants to lipopolysaccharides (LPS) + interferon-γ [IFN-γ) is an injury model that recapitulates the key hallmarks of inflammatory CNS diseases, which include demyelination, glial reactivity, and neuronal death [13,14]. By mimicking the interaction of microglia with infiltrating immune T cells, the insult triggers the release of proinflammatory and cytotoxic factors and promotes inflammatory neurodegeneration [14]. Recently, we showed that the exposure of hippocampal explants to LPS + IFN-γ-induced a selective neurodegeneration in the CA1 pyramidal layer that can be monitored by propidium iodide uptake, thus enabling the study of the neuroprotective effects of pharmacological agents [13]. Here, we asked whether this model could also reproduce the glial response to demyelination, thus enabling the study of the effect of compounds on myelin clearance under neuroinflammation. 

The observation that myelin damage is generated early under LPS + IFN-γ exposure, and that it precedes the abrupt appearance of neuronal death occurring after 4 days [13], gave us a unique opportunity to explore the contribution of glial cells to myelin internalization under neuroinflammation during the 3 days before the onset of massive neurodegeneration. To accomplish our aim, we employed high resolution Z-stack confocal laser-scanning microscopy to investigate the coexpression between myelin basic protein (MBP) and oligodendrocyte transcription factor 2 (Olig2), nerve-glial antigen 2 (NG2), glial fibrillary acidic protein (GFAP), and ionized calcium-binding adapter molecule 1 (Iba1) markers in hippocampal explants exposed to LPS + IFN-γ for 3 days.

By demonstrating a time-dependent response of oligodendrocyte lineage, microglia, and astrocytes to inflammatory demyelination, our findings indicate that hippocampal explant exposure to LPS + IFN-γ is a valuable demyelination model with which to reproduce extrinsic and intrinsic myelin uptake by glial cells under neuroinflammation. Moreover, our data provide evidence of a marked microglial response and suggest that the cross-talk between microglia and oligodendrocytes may be relevant to the neurodegeneration progression in demyelinating diseases.

## 2. Materials and Methods

### 2.1. Animals

All animal experiments, handling, and care were in accordance with ARRIVE guidelines and the Guide for the Care and Use of Laboratory Animals (EU Directive 2010/63/EU). The protocol was approved by the Animal Care and Use Committee of “Federico II” University of Naples, Italy, and Ministry of Health, Italy (#515/2019-PR). Pregnant female Wistar rats were obtained from Charles River Laboratories, Calco, (Italy) and maintained at a constant temperature (22 + 1 °C) on a 12-h light/dark cycle (lights on at 7 AM) with food and water ad libitum. P7–P9 littermates were used to make organotypic explants. All efforts were made to minimize animal suffering and reduce the number of animals. Both male and female animals were used for organotypic explant preparation.

### 2.2. Hippocampal Organotypic Explants

Organotypic explants were made as previously described [13,15,16] by postnatal P7- P9-day-old Wistar rat pups (Charles River Laboratories). Parasagittal slices (400 μm thick) obtained from hippocampi using a McIlwain tissue chopper (Campden Instruments, Leicester, UK) were transferred to a humidified semiporous membrane (Millicell inserts of 0.4 mm pore size, Merck-Life Science S.r.l., Milan, Italy) in six-well tissue culture plates (4–5 slices per membrane). The slices were cultured in normal medium (NM) consisting of 50% minimal essential medium (MEM, Thermo Fisher Scientific, Monza, Italy), 25% Hank’s balanced salt solution (HBSS, Thermo Fisher Scientific, Monza, Italy), 25% heat inactivated horse serum (HS, Thermo Fisher Scientific, Monza, Italy), 6.5 mg/mL glucose, 1 mM glutamine, and 1.5% fungizone (Thermo Fisher Scientific, Monza, Italy) Cultures were maintained at a 37 °C and 5% CO_2_-conditioned atmosphere. Experiments were performed on cultures kept in vitro for 10–12 days (10–12 DIV).

### 2.3. LPS Plus INF-γ-Exposure

LPS plus INF-γ exposure was performed in hippocampal cultures as already described [13]. Explants were removed from NM medium, washed in low-serum medium (LSM) (consisting of NM with serum replaced with MEM, plus 1% HS), and exposed to 10 μg/mL LPS (Merck-Life Science S.r.l., Milan, Italy) plus 100 ng/mL INF-γ (Thermo Fisher Scientific, Monza, Italy) for 3 days in LSM.

### 2.4. Confocal Microscopy 

Confocal analysis was performed as previously described [17,18,19]. After fixation and blocking, slices were incubated with the indicated primary antibodies for 48 h at 4 °C: rat monoclonal anti-MBP (1:1000, Merck-Life Science S.r.l., Milan, Italy), rabbit polyclonal anti-Olig2 (1:200, Merck-Life Science Merck-Life Science S.r.l., Milan, Italy), rabbit polyclonal anti-NG2 (1:400, Merck-Life Science S.r.l., Milan, Italy), rabbit polyclonal anti-Iba1 (1:2000, FUJIFILM Wako Chemicals, Osaka, Japan), rabbit polyclonal anti-GFAP (1:1000, Merck-Life Science, Merck-Life Science S.r.l., Milan, Italy), and mouse monoclonal anti-low density lipoprotein receptor-related protein 1 (LRP1, 1:200 Santa Cruz Biotechnology, Inc., Dallas, TX, USA). Then, slices were incubated with the corresponding fluorescence- labeled secondary antibodies (Alexa488- or Alexa594-conjugated anti-mouse, anti-rabbit or anti-rat IgGs, Molecular Probes, Thermo Fisher Scientific, Monza, Italy) for 2 h at room temperature. Finally, they were mounted on glass slides and imaged using a Zeiss LSM 700 laser scanning confocal microscope (Carl Zeiss, Jena, Germany).

Method controls included replacement of the primary antisera with normal serum (1:200). To control for a possible cross-reactivity between IgGs in double immunolabeling experiments, some sections were processed through the same immunocytochemical sequence except that either the primary antisera were replaced with normal serum or only one primary antibody was applied; however, the full complement of secondary antibodies was maintained. In addition, the secondary antibodies utilized were highly pre-adsorbed to the IgGs of numerous species. Tissue labeling without primary antibodies was also tested to exclude autofluorescence. No specific staining was observed under these control conditions, thus confirming the specificity of the immunosignals.

### 2.5. Quantification of Confocal Studies 

Stacks of photographs of NG2/MBP, Iba1/MBP, and GFAP/MBP immunolabeling at 2 μm intervals in the CA1 region were obtained as previously described [13]. Images (4-5) of randomly chosen areas were acquired with identical intensity of fluorescence. Maximum intensity projection (MIP) images were constructed by using ZEN imaging software. The quantification of co-localization between Iba1 and MBP, GFAP and MBP, and NG2 and MBP immunostaining was determined by using ImageJ software (NIH, Bethesda, MD, USA) as described [18]. Images were thresholded first, and then the number of pixels positive for NG2/MBP, Iba1/MBP, and GFAP/MBP was calculated. This value was expressed as a percentage of co-localization and indicated the amount of NG2/MBP, Iba1/MBP, and GFAP/MBP overlapping.

The number of NG2^+^, Iba1^+^, and GFAP^+^ cells with internalized myelin particles was estimated by the respective total numbers of positive cells found using double-labeled cells in MIP views, which were created from stacks and normalized.

Double labeling with Olig2 and MBP antibodies was used to investigate MBP distribution in oligodendrocytes of the CA1 subfield. Individual MBP^+^/Olig2^+^ cells were scored according to somatic MBP immunoreactivity in 3 main categories: (i) peri-MBP^+^, displaying scarce or moderate MBP immunoreactivity at peri-membrane sites; (ii) peri-MBP^++^, displaying an intense MBP immunosignal at peri-membrane sites; (iii) cyto-MBP, displaying pronounced MBP immunoreactivity within the cytosol. 

To characterize the demyelination process in hippocampal explants under neuroinflammation, the number of intact myelin segments and the accumulation of myelin debris clusters deriving from fragmented myelin segments were measured at different time points after LPS + IFN-γ exposure. To identify debris in tissue slices, a binary mask on MBP immunosignal was created using ImageJ software. Particle analysis was used to identify particle-like MBP^+^ areas or blobs in the extracellular space of tissue slices. To exclude background particles, an analysis of particle criteria was applied to automatically count all debris. The lower limit of the particle area was set to 70 pixels. MBP+ cells, including those with MBP immunoreactivity at peri-membrane sites, were excluded from the images. The number of MBP^+^ positive segments were counted manually and quantified per mm^2^. All analyses were performed blinded to sample identity.

To analyze the early role of oligodendrocyte lineage in myelin uptake we performed double immunofluorescence experiments with antibodies against Olig2 and the low density lipoprotein receptor-related protein-1 (LRP1) at 1 day after LPS + IFN-γ exposure. The number of NG2^+^ and double-labeled Olig2^+^-LRP1^+^ cells was assessed in the CA1 area via manual counting at 40× magnification. Only cells with a clearly visible cell soma were counted.

NG2 immunofluorescence was quantified in terms of pixel intensity using NIH image software as previously described [20,21,22]. Digital images were taken with a 40× objective lens, and identical laser power settings and exposure times were applied to all photographs from each experimental set.

### 2.6. Statistical Analysis

Using randomization, the experiments were conceived to generate groups of equal size,. Experiments were performed on 15–20 explant slices from at least three independent litters. Results obtained for each pup were averaged and used as the experimental unit in statistical analyses. Sample sizes are indicated in the figure legends. Both sample processing and quantification in microscopy studies were performed in a blinded manner. In some experiments, data were normalized to reduce unwanted sources of variation, as is also indicated in the figure legends. The data are expressed as the mean ± SEM of the values obtained from individual experiments. Statistical comparisons between two groups were performed using two-tailed Student’s t tests. Statistical comparisons for multiple groups were performed using one-way analysis of variance (ANOVA) followed by Bonferroni post hoc analysis. GraphPad Prism 6.0 was used for statistical analysis (GraphPad Software, Inc, La Jolla, CA, USA). Post hoc tests were conducted only if F in ANOVA achieved *p* < 0.05. * *p* < 0.05 was considered the threshold for statistical significance.

## 3. Results

### 3.1. LPS + IFN-γ Exposure-Induced Myelin Damage in the CA1 Region of Hippocampal Explant Cultures

**Figure 1:**
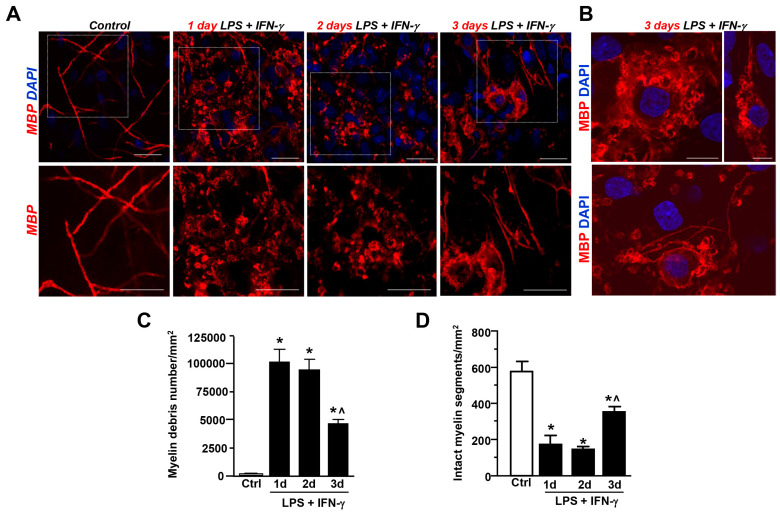
LPS + IFN-γ-induced demyelination in the CA1 region of rat hippocampal organotypic slices. (**A**) Representative confocal images of MBP immunostaining in rat hippocampal slices under control conditions and after LPS + IFN-γ-treatment for 1, 2, and 3 days. The box diagram is displayed at higher magnification in the lower panels. Scale bars: 20 μm. (**B**) Confocal images showing higher magnification of MBP^+^ cells observed after 3 days of LPS + IFN-γ. Scale bars: 10 μm. Nuclei are stained with DAPI (blue). C-D, Quantitative analysis of myelin debris accumulation (**C**) and intact myelin segments (**D**) under control conditions and after LPS + IFN-γ treatment for 1, 2, and 3 days. Data are expressed as the mean number of myelin debris or intact segments ± SEM per unit area (mm^2^) (n = 3). In (**C**), * *p* < 0.05, 1 day, 2 days, and 3 days versus control; ^˄^ *p* < 0.05, 3 days versus 1 day and 2 days. In (**D**), * *p* < 0.05, 1 day, 2 days, and 3 days versus control; ^˄^ *p* < 0.05, 3 days versus 2 days.

We have previously shown that the exposure of organotypic explant cultures to LPS + IFN-γ for 3 days decreased MBP levels in the CA1 region, thus indicating myelin damage resulting from neuroinflammatory insult [13]. As shown in Figure 1A, the neuroinflammatory insult induced a drastic reduction in intact myelin segments at day 1 and day 2 after insult. Quantitative analyses revealed a massive accumulation of MBP^+^ debris in the CA1 hippocampal subfield after 1 and 2 days, while accumulation significantly decreased after 3 days (Figure 1C). In parallel, a significant increase in the number of MBP^+^ segments with a thin and scattered appearance was observed after 3 days when compared to days 1 and 2 (Figure 1B,D). Interestingly, MBP^+^ oligodendrocytes with abundant and foamy cytoplasm were also observed 3 days after insult (Figure 1B). These findings suggest that myelin debris uptake and clearance is an active process in hippocampal explants exposed to LPS + IFN-γ-induced neuroinflammation.

### 3.2. Contribution of Olig2^+^ and NG2^+^ Cells to Myelin Uptake after LPS + IFN-γ-Exposure in Hippocampal Explants

**Figure 2:**
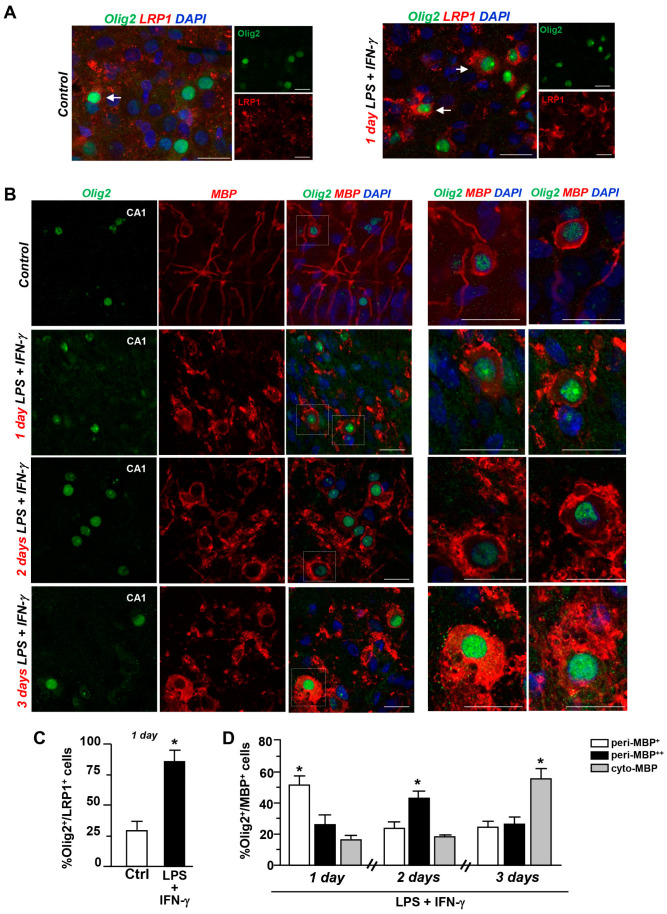
Coexpression of Olig2 with LRP1 and MBP in the CA1 region during LPS + IFN-γ-induced demyelination. (**A**) Confocal images displaying coexpression of Olig2 (green) and LRP1 (red) in explants under control conditions and after LPS + IFN-γ treatment for 1 day. Nuclei are stained with DAPI (blue). Arrows point to intensely stained Olig2^+^/LRP1^+^ cells at 1 day. Scale bars: 20 μm. B, left panels, Representative confocal images showing Olig2 (green) and MBP (red) coexpression under control conditions and after LPS + IFN-γ exposure for 1, 2, and 3 days. (**B**) right panels, Higher magnification images displaying representative double-labeled Olig2^+^/MBP^+^ cells observed at day 1, day 2, and day 3. Note the accumulation of MBP immunoreactivity at peri-membrane soma sites of Olig2^+^ cells at day 2 and within the cytosol at day 3. Scale bars: 20 μm. (**C**) Quantitative analysis of double-labeled LRP1^+^/Olig2^+^ cells in slices under control conditions and after LPS + IFN-γ treatment for 1 day. The values represent the mean ± S.E.M. (n = 3). * *p* < 0.05, LPS + IFN-γ versus control. (**D**) Quantitative analysis of the number of double-labeled Olig2^+^/MBP^+^ scored according to somatic MBP immunoreactivity at day 1, day 2, and day 3. The values represent the mean ± S.E.M. (n = 3). * *p* < 0.05, peri-MBP^+^ versus peri-MBP^++^ and cyto-MBP at 1 day; * *p* < 0.05, peri-MBP^++^ versus peri-MBP^+^ and cyto-MBP at 2 days; * *p* < 0.05, cyto-MBP versus peri-MBP^+^ and peri-MBP^++^ at 3 days.

Quantitative analysis revealed a significant increase in the number of Olig2^+^ cells expressing LRP1 (about 80%), a major receptor for internalization of degraded myelin [23], when compared to untreated controls (Figure 2A,C). Then, to explore the subcellular distribution of MBP in oligodendrocytes under control and neuroinflammation conditions, respectively, we performed double immunofluorescence analysis with anti-Olig2 and anti-MBP antibodies. Individual scoring of Olig2^+^ cells according to somatic MBP distribution revealed that double-labeled Olig2^+^/MBP^+^ cells accumulated MBP immunoreactivity at peri-plasma membrane sites after 1 day of LPS + IFN-γ-treatment, and did so even more after 2 days. On day 3, we observed a significant increase in the number of Olig2^+^ cells with intense cytoplasmic MBP immunoreactivity, which somehow confers a foamy appearance to Olig2^+^ cells (Figure 2B,D).

**Figure 3:**
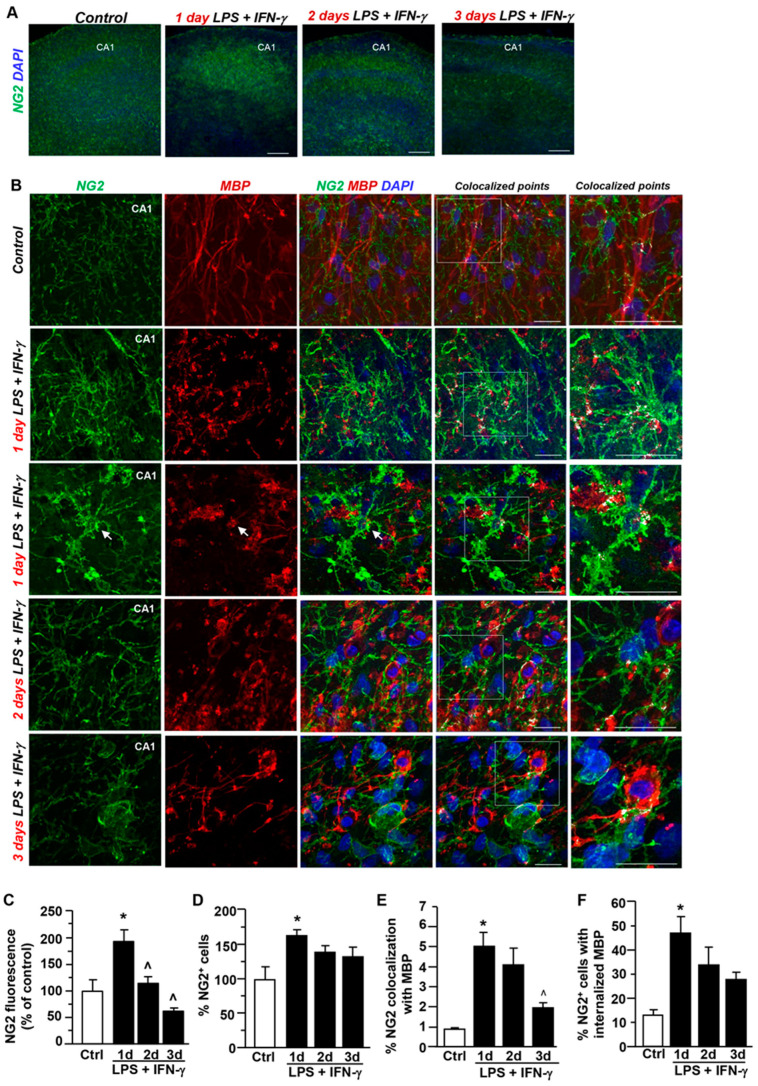
Coexpression of NG2 with MBP in the CA1 region during LPS + IFN-γ-induced demyelination. (**A**) Low magnification confocal images of NG2 immunostaining in explants under control conditions and after exposure to LPS + IFN-γ for 1, 2, and 3 days, respectively. Scale bars: 200 μm (**B**) MIP images displaying NG2 (green) and MBP (red) coexpression under control conditions and after LPS + IFN-γ exposure for 1, 2, and 3 days. Nuclei are stained with DAPI (blue). White pixels display the colocalized points. The box diagram in colocalized points is shown at higher magnification. Arrows point to colocalizing cells. Scale bars: 20 μm. (**C**,**D**) Densitometric analysis of NG2 fluorescence intensity (**C**) Quantitative analysis of the number of NG2+ cells (**D**) in hippocampal cultures under control conditions and after LPS + IFN-γ exposure for 1, 2, and 3 days. Data are expressed as percentage of control. * *p* < 0.05, one day versus control. ^˄^ *p* < 0.05, 2 days and 3 days versus 1 day. (**E**) Quantitative analysis of NG2 colocalization with MBP immunosignal in slices under control conditions and after LPS + IFN-γ treatment for 1, 2, and 3 days. The values represent the mean ± S.E.M. (n = 3). * *p* < 0.05, 1 day versus control. ^˄^ *p* < 0.05, 3 days versus 1 day. (**F**) Quantitative analysis of the number of NG2+ cells displaying MBP droplets in slices under control conditions and after LPS + IFN-γ treatment for 1, 2, and 3 days. The values represent the mean ± S.E.M. (n = 3). * *p* < 0.05, 1 day versus control.

Then, to discriminate the role of oligodendrocyte precursors we analyzed the coexpression of the NG2 marker with MBP after LPS + IFN-γ exposure. Neuroinflammatory insult significantly increased NG2 immunofluorescence intensity in the CA1 region at 1 day; then, it declined and returned to basal levels after 3 days (Figure 3A–C). Analysis of cell number revealed an upregulated number of NG2^+^ cells 1 day after neuroinflammatory insult in the most vulnerable CA1 region (Figure 3D). The coexpression of NG2 with MBP significantly increased after LPS + IFN-γ treatment for 1 day, while a decline versus basal levels was measured after 3 days (Figure 3A,E). The early overlapping signal was found not only at NG2/MBP contact sites, but also within processes or soma of NG2^+^ cells, as revealed by the increased number of cells displaying MBP^+^ particles after 1 day (about 45%) (Figure 3A,F). These findings suggest an active contribution of oligodendrocyte lineage cells to myelin uptake in hippocampal explants early after LPS + IFN-γ-exposure.

### 3.3. Contribution of Astrocytes and Microglia/Macrophages to Myelin Uptake after LPS + IFN-γ Exposure in Hippocampal Explants

Next, to investigate the contribution of astrocytes and microglia to myelin uptake after LPS + IFN-γ exposure, 10–12 DIV organotypic explants were exposed to LPS + IFN-γ; the coexpression of MBP with GFAP or Iba1 was analyzed after insult.

**Figure 4:**
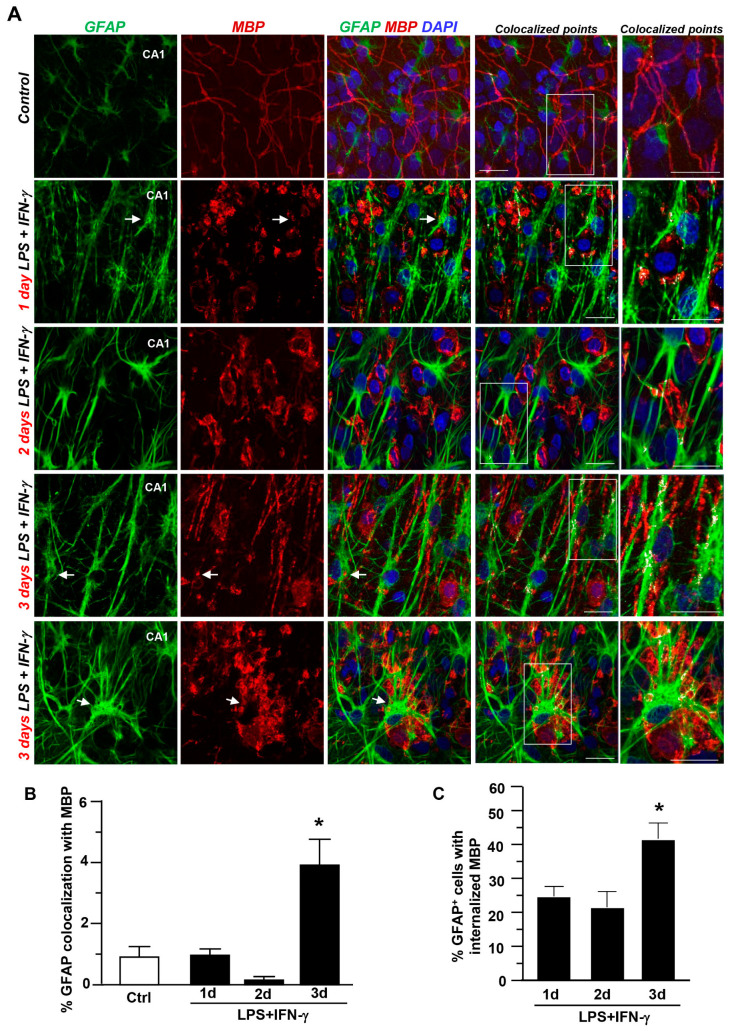
Coexpression of GFAP with MBP in the CA1 region during LPS + IFN-γ-induced demyelination. (**A**) MIP confocal images showing GFAP (green) and MBP (red) coexpression under control conditions and after LPS + IFN-γ exposure for 1, 2, and 3 days. Nuclei are stained with DAPI (blue). Colocalization is shown by white pixels. The box diagram in colocalized points is shown at higher magnification. Arrows point to colocalizing cells. Scale bars: 20 μm. (**B**) Quantitative analysis of GFAP colocalization with MBP immunosignal under control conditions and following LPS + IFN-γ treatment for 1, 2, and 3 days. The values represent the mean ± S.E.M. (n = 3). * *p* < 0.05, 3 days versus all groups. (**C**) Quantitative analysis of the number of GFAP^+^ astrocytes with internalized MBP particles after LPS + IFN-γ exposure for 1, 2, and 3 days. Data are expressed as a percentage of the total number of GFAP^+^ cells. * *p* < 0.05, 3 days versus all groups.

**Figure 5:**
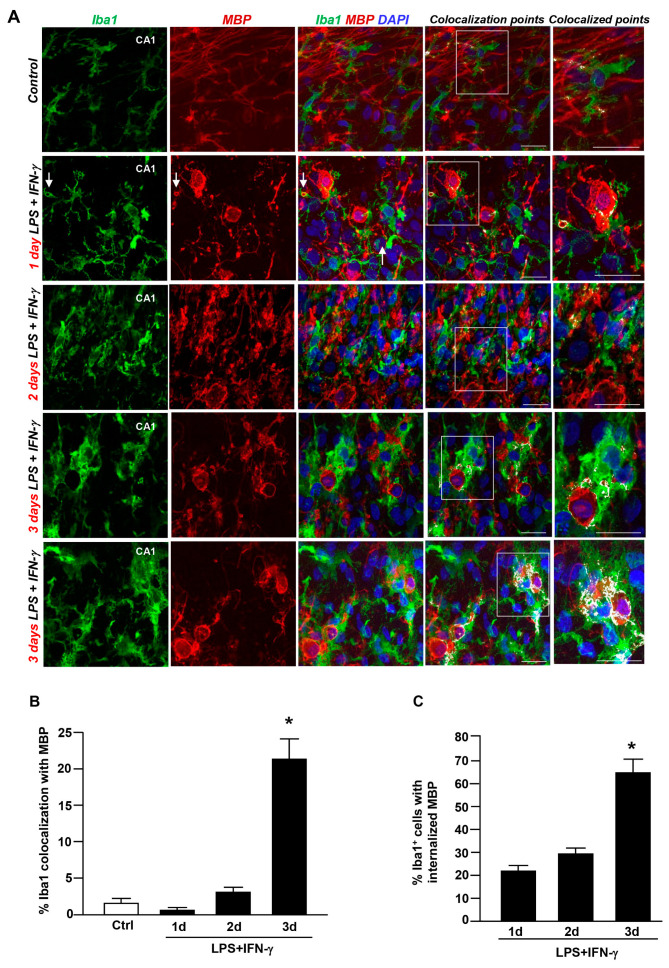
Coexpression of Iba1 with MBP in the CA1 region during LPS + IFN-γ-induced demyelination. (**A**) MIP images displaying Iba1 (green) and MBP (red) coexpression under control conditions and after LPS + IFN-γ exposure for 1, 2, and 3 days. Nuclei are stained with DAPI (blue). Colocalization is shown by white pixels. The box diagram in colocalized points is shown at higher magnification. Arrows point to colocalizing cells. Scale bars: 20 μm. (**B**) Quantitative analysis of Iba1 colocalization with MBP immunosignal in slices under control conditions and following LPS + IFN-γ treatment for 1, 2, and 3 days. The values represent the mean ± S.E.M. (n = 3). * *p* < 0.05, 3 days versus all groups. (**C**) Quantitative analysis of the number of Iba1^+^ microglia with internalized MBP particles after LPS + IFN-γ exposure for 1, 2, and 3 days. Data are expressed as percentage of the total number of Iba1^+^ cells. * *p* < 0.05, 3 days versus all groups.

Confocal double immunofluorescence analysis revealed scarce coexpression of GFAP and Iba1 with MBP in both control slices and those at 1 and 2 days after inflammatory insult (Figure 4A,B and Figure 5A,B, respectively). Similarly, quantitative analysis revealed about 20% of GFAP- and Iba1-expressing cells with accumulated MBP^+^ particles in the cytosol (Figure 4C and Figure 5C, respectively).

By contrast, a marked increase in Iba1 colocalization with MBP occurred 3 days after insult, when about 80% of microglia/macrophages accumulated MBP^+^ debris within the cytosol (Figure 5C). To a much more limited extent, quantitative assessment revealed an increased GFAP coexpression with MBP after 3 days, with about 40% of GFAP^+^ cells displaying internalized MBP^+^ particles (Figure 4C). Interestingly, Z-stack analysis performed 3 days after insult revealed that Iba1^+^ macrophages largely overlapped with the soma of several MBP^+^ cells in hippocampal explants (Figure 5A), indicating the engulfment of whole MBP^+^ cells by microglia. These findings highlight the major role of microglia/macrophages in myelin processing under neuroinflammation in hippocampal slices.

## 4. Discussion

In the present study we showed that the exposure of hippocampal explants to LPS plus IFN-γ, a cytokine produced by auto-antigen-reactive T cells in multiple sclerosis, induced neuroinflammatory demyelination in the CA1 region and a time-dependent response of the glial scar to myelin damage and uptake. Oligodendrocyte precursors internalized MBP particles early after insult, while mature oligodendrocytes progressively increased MBP immunoreactivity both at peri-membrane sites and within the cytosol. Microglia/macrophages contributed greatly to myelin uptake under neuroinflammation in hippocampal explants, while less contribution was exerted by GFAP^+^ astrocytes. 

Previous studies from our research group and others have shown that by mimicking the microglia interaction with infiltrating immune T cells, the application of LPS + IFN-γ in hippocampal explants increased microglia and astrocyte reactivities, and promoted an inflammatory demyelinating insult with massive neurodegeneration [13,14]. We recently showed that neuronal vulnerability in hippocampal slices exposed to LPS + IFN-γ exposure is influenced by the ex vivo maturation stage [13]. This finding may explain an apparent discrepancy in our study, in which massive neurodegeneration was observed in the CA1 region of 10–12 DIV cultures after 4 days [13], as compared to others in which it was detected in both the CA1 and CA3 subfields of 7 DIV cultures after 3 days [14].

Here we showed that myelin debris accumulation in 10–12 DIV hippocampal explants exposed to LPS + IFN-γ started immediately after insult in the most vulnerable CA1 region and declined after 3 days, a time point characterized by the re-appearance of several thin myelinated segments, suggesting a tentative repair process. In line with this observation, it has been shown that myelin repair occurs in the presence of concurrent inflammation in both experimentally induced and clinical demyelination (i.e., in MS lesions). Furthermore, surviving mature oligodendrocytes can both extend processes and unsheathe demyelinated axons in a hostile environment [24,25].

Our morphological studies revealed that the expression of NG2, a marker of OPCs, increased both early and transiently in the CA1 region at 1 day, and that it was accompanied by a concomitant increase in the number of NG2^+^ cells that subsequently declined. Corroborating these findings, NG2 expressing cells have been shown to proliferate and accumulate in the glial scar after injury or in response to myelin debris exposure [26]. We also found that NG2^+^ cells accumulated MBP^+^ particles along processes and within the soma, possibly suggesting a role for the cytosol in phagocytic digestion. These results are in line with recent transcriptome analyses showing that OPCs can both adopt immune-competent states in the context of inflammatory demyelination and express genes that encode proteins with roles in phagocytosis [9,27,28]. Consistent with this role, previous findings indicated that OPCs may engulf neuronal material in the developing and healthy brain [29,30,31,32]. In accordance with the role of oligodendrocyte lineage in myelin uptake and processing, our coexpression analyses showed that the LRP1 receptor, an essential endocytic receptor for myelin internalization [23], was abundantly co-expressed by Olig2^+^ cells at day 1 after inflammatory insult, suggesting an MBP endocytic activity in oligodendrocyte lineage early after demyelination. Consistent with this result, we found that Olig2^+^ oligodendrocytes in the CA1 region accumulated MBP^+^ material at peri-membrane sites, suggesting the contribution of oligodendrocytes to myelin uptake under neuroinflammation. Although further studies are needed to better characterize the fate of myelin in oligodendrocyte lineage under neuroinflammation, previous studies have shown that autophagy in oligodendrocytes is essential for myelin maintenance, and that its depletion is detrimental to oligodendrocyte function after spinal cord injury [33,34,35]. Moreover, recent works clearly showed that retractions of entire myelin sheaths during development involved both the uptake of myelin and lysosomal degradation by oligodendrocytes [36]. 

In support of the well-recognized role of microglia/macrophages in myelin debris removal under neuroinflammation [4,37,38], we found a dramatic increase in MBP coexpression with microglia/macrophages 3 days after insult, a time point characterized by the significant decline of myelin debris in the CA1 region. At this time point, Iba1^+^ cells displayed accumulated MBP^+^ material within the cytosol; more interestingly, these cells engulfed whole MBP^+^ cells. Recent data have provided evidence that microglia phagocytose OPCs as a homeostatic mechanism for proper myelination during early postnatal development [39]. Conversely, macrophage attack on oligodendrocytes under ischemic conditions has been shown to prevent endogenous oligodendrocyte replacement and repair responses [40]. The observation that microglia-engulfed actions on MBP^+^ cells preceded the appearance of neuroinflammatory damage in our explant model [13] seems to support the detrimental role of microglia attack to oligodendrocytes in recovery response. 

A recent study indicated that the phagocytic activity of astrocytes may compensate for dysfunctional activity of microglia [41]. The minor involvement of astrocytic-mediated myelin uptake observed in our study, when compared to that of microglia, may support this hypothesis.

## 5. Conclusions

Collectively, our findings indicate that hippocampal explant exposure to LPS + IFN-γ-induced neuroinflammation may represent a valuable demyelination model for studying treatment strategies modulating myelin uptake and clearance by glial scar. In addition, beside improving our understanding of the spatial-temporal contribution of glial cells to myelin uptake under neuroinflammation, our data reveal the crucial response of microglia and oligodendrocytes to demyelination. Of particular relevance, this work suggests that microglia-oligodendrocytes cross-talk under neuroinflammation may be critical for neurodegeneration progression. Our findings may therefore be relevant for brain diseases featuring demyelination, such as multiple sclerosis and Alzheimer’s disease.

## Data Availability

The data presented in this study are available on request from the corresponding author.
